# Transcriptome Analysis and Expression of Selected Cationic Amino Acid Transporters in the Liver of Broiler Chicken Fed Diets with Varying Concentrations of Lysine

**DOI:** 10.3390/ijms21165594

**Published:** 2020-08-05

**Authors:** Collins N. Khwatenge, Boniface M. Kimathi, Samuel N. Nahashon

**Affiliations:** 1 Department of Biological Sciences, Tennessee State University, Nashville, TN 37209, USA; collinskhwatenge@yahoo.com (C.N.K.); muthurikim@gmail.com (B.M.K.); 2 Department of Agricultural and Environmental Sciences, Tennessee State University, Nashville, TN 37209, USA

**Keywords:** lysine, broiler chickens, growth performance, cationic amino acid transporters, RNA-seq, transcriptome analysis, differentially expressed genes

## Abstract

Amino acids are known to play a key role in gene expression regulation. Amino acid signaling is mediated via two pathways: the mammalian target of rapamycin complex 1 (mTORC1) and the amino acid responsive (AAR) pathways. Cationic amino acid transporters (CATs) are crucial in these pathways due to their sensing, signaling and transport functions. The availability of certain amino acids plays a key role in the intake of other amino acids, hence affecting growth in young birds. However, the specific mechanism for regulating lysine transport for growth is not clear. In this study, we analyze the transcriptome profiles and mRNA expression of selected cationic amino acid transporters in the livers of broilers fed low and high lysine diets. Birds consumed high-lysine (1.42% lysine) or low-lysine (0.85% lysine) diets while the control group consumed 1.14% lysine diet. These concentrations of lysine represent 125% (high lysine), 75% (low lysine) and 100% (control), respectively, of the National Research Council’s (NRC) recommendation for broiler chickens. After comparing the two groups, 210 differentially expressed genes (DEGs) were identified (fold change >1 and false discovery rate (FDR) <0.05). When comparing the high lysine and the low lysine treatments, there were 67 upregulated genes and 143 downregulated genes among these DEGs. Analysis of Kyoto Encyclopedia of Genes and Genomes (KEGG) and the Gene Ontology (GO) enrichment analysis show that cellular growth, lipid metabolism and lysine metabolism pathways were among the significantly enriched pathways. This study contributes to a better understanding of the potential molecular mechanisms underlying the correlation between lysine intake, body weight gain (BWG) and feed intake (FI) in broiler chickens. Moreover, the DEGs obtained in this study may be used as potential candidate genes for further investigation of broiler growth customized responses to individualized nutrients such as amino acids.

## 1. Introduction

Lysine is an essential and one of the most limiting amino acids for growth. Amino acids are generally required for muscle development [1]. Lysine is required for protein synthesis and muscle deposition, synthesis of cytokines, gene expression and lymphocyte proliferation and thus in the optimum functioning of immune system in response to infection. Therefore, an inadequate supply of lysine would lead to reduced growth and a reduction in antibody response and cell-mediated immunity in chickens [2]. Several studies have shown that an increase in supplementary lysine in feed increases the performance of growing birds [3,4,5,6]. In the case of broilers, it has been shown that there is a negative impact of imbalance and antagonism among amino acids on nutrient intake and utilization. Therefore, care should be exercised while developing effective strategies for feed formulation in order to obtain maximum utilization of amino acids.

The ingestion of nutrients such as amino acids as a signal induces transcription, RNA processing and stability, protein synthesis and modification. These processes affect DNA replication and regulation of gene expression in mammalian and avian cells [7]. Therefore, supplementation or lack of exogenous amino acids can effectively regulate gene expression in animals [8].

Lysine catabolism is principally located in the liver [9]. Many studies suggest that lysine degradation takes place ultimately in two distinct routes, the pipecolic acid and saccharopine routes. These two routes converge ultimately at the α-aminoadipic semialdehyde level [10]. Therefore, the liver plays an important role, including in the sensing of other amino acids [11]. Amino acid transporters such as cationic amino acid transporters (CATs) play an important role in the sensing of amino acid levels, both directly as initiators of nutrient signaling and indirectly as regulators of external amino acid access to intracellular receptor/signaling mechanisms [12]. CATs belong to the solute carrier (SLC) family, which is divided into two subgroups: the cationic amino acid transporters (CATs, SLC7A1-4 and SLC7A14) and the light or catalytic subunits (L-type amino acid transporters (LATs), SLC7A5-13 and SLC7A15) of the heteromeric amino acid transporters (HATs) [13]. Signaling intermediates between nutrient availability and altered gene expression are vital in determining ways to improve production performance of poultry, hence reflecting the status of animal growth and development.

Research into the mechanisms of molecular regulation of lysine utilization is key to understanding the molecular mechanisms of lysine degradation and biosynthesis for the maximization of growth performance in broiler chicken. Here, we used RNA sequencing to profile liver transcriptome and thereby identify the transcriptional variations and biological pathways between broiler chickens fed low-lysine and high-lysine diets (75% and 125% of National Research Council (NRC) requirements, respectively). In a broader sense, a transcriptome consists of a protein-coding messenger RNA (mRNA) and non-coding RNA (ncRNA), ribosomal RNA (rRNA), transfer RNA (tRNA) and other ncRNAs [14,15]. In a narrow sense, it refers to the collection of all mRNAs that can be applied in the study of poultry transcriptomes to study the molecular mechanisms as well as gene structure and functions of avian species’ responses to amino acids and other nutritional changes [16,17,18,19,20,21,22,23,24,25,26].

To the best of our knowledge, this is the first study which employs transcriptome analysis to investigate the molecular mechanisms and identification of lysine-dependent candidate genes to be used as main targets for improving the growth performance and production of broiler chickens. Our findings may guide the exploration of potential ways for improving the growth and production performance of broiler chickens by examining the resultant theoretical molecular mechanism of a gene-to-lysine interaction level. Moreover, the results of this study can be used to directly link individual amino acids with key metabolic pathways and the improvement of utilization of lysine and other amino acids to improve growth in poultry.

## 2. Results

### 2.1. Growth Performance

**Figure 1:**
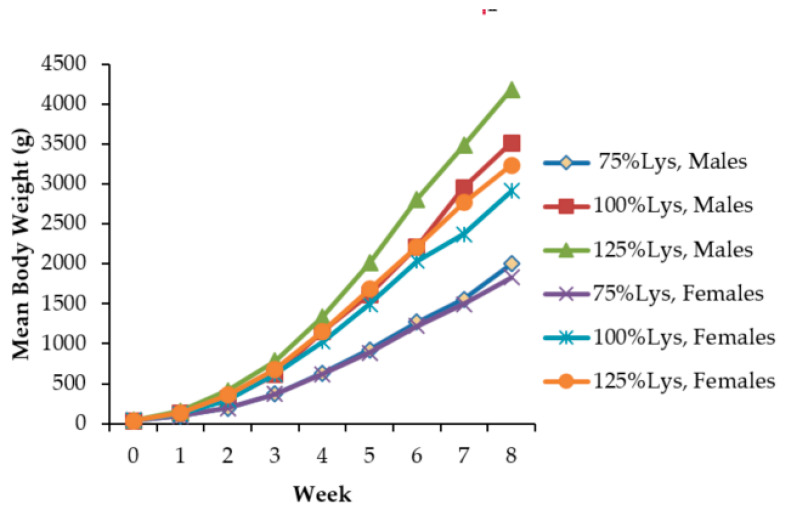
Effects of lysine on mean body weight. There is a significant increase in body weight with an increase in lysine concentration from week 1 to week 8. Male broilers had higher mean body weights on average than the female broilers in each ration. Lys: dietary lysine.

Lysine x sex interactions were not significant (*p* > 0.05); therefore, mean growth performance data for males and females were pooled. The responses of broiler chickens to varying concentrations of dietary lysine are presented in Table 1. The effect of dietary lysine on the growth rate of the broilers was similar across the three lysine treatments. There was a significant increase in body weight with an increase in lysine concentration from week 1 to week 8 (Figure 1). The body weight, feed intake, body weight gain (BWG) and lysine consumption of low-lysine (lysine-deficient) birds were lower than those of high-lysine (lysine-adequate) birds and control chicks (*p* < 0.05). The average feed conversion ratio (FCR) and percentage mortality of low-lysine birds was significantly higher (*p* < 0.05) than the birds on the control and the high-lysine diets. There was, however, no significant difference in FCR and percentage mortality between birds fed the control lysine concentrations and the high lysine (*p* < 0.05).

### 2.2. Sequencing Data and Quality Control

After the transcriptional analysis of the low-lysine versus high-lysine samples, 16.83 million clean reads were obtained by the quality control. The clean reads from each sample reached above 2.8 million; the guanine-cytosine (GC) content was between 44.76% and 52.46%. The percentage of final library in each sample of sequenced reads was greater than 51% (Table 2). The distribution of the mapped reads in different regions of the chicken reference genome showed that most of the reads had been mapped to the coding sequence of exons and introns. Total fragments in the introns were 15.88%–37.55%: 40%–65.59% were in the exons, and 11%–48.99% were intergenic. The above results indicate that the data were reliable enough to be used for further analysis. The RNA-seq reads from the livers of chickens fed diets containing 75% and 125% of the NRC lysine requirement were deposited in the NCBI Sequence Read Archive (SRA) (Accession No. PRJNA625635).

### 2.3. Gene Expression Profile and Differential Expression Analysis

**Figure 2:**
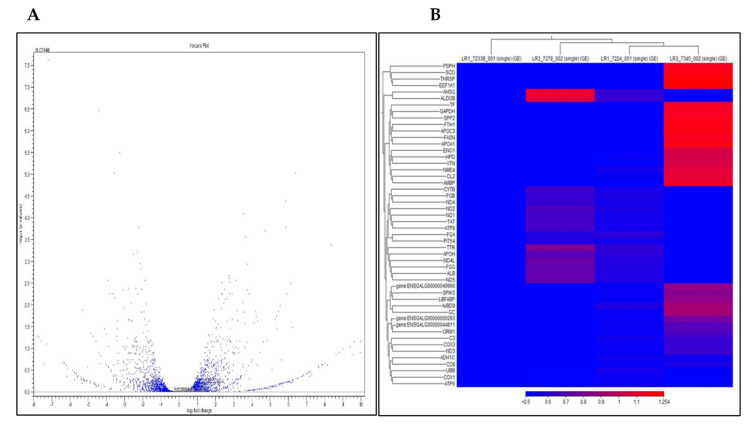
Expression profiles of differentially expressed genes (DEGs) in lysine-deficient and lysine-adequate treatment groups of broiler chickens. (**A**) Volcano plot of DEGs between the high and low lysine groups. (**B**) Heatmap of the expression levels of DEGs in liver tissues. The color scale below the heatmap represents log10 expression values. Red and blue colors indicate higher and lower levels, respectively.

Gene expression abundance was measured by the fragments per kilobase of transcripts per million mapped fragments (FPKM) and biological replicates of both treatments showed a similar FPKM distribution. The 75% lysine and 125% lysine treatment groups were chosen since they are divergent in lysine concentrations are suitable for transcriptome analysis. The results of merging the assembled transcripts in the two sets of sample treatments exhibited a total of 12,631 genes being expressed in both treatments. Differential gene expression analysis was conducted to characterize the transcriptional variations occurring between the liver tissues from broiler chickens fed low and high lysine concentrations. There was a total of 210 differentially expressed genes (DEGs) that were identified by comparing gene expression between the two treatment groups (fold change, FC > 2 and False Discovery Rate, FDR < 0.05). A comparison of RNA-seq data between the low lysine treatment group (LR1-7233B, LR1-7224, LR1-7233) and the high lysine treatment group (LR3-7278, LR3-7345, LR3-7320) revealed that 99 genes were upregulated and 85 genes were downregulated among these DEGs. The volcanic map (volcano plot) obtained from the RNA-seq analysis shows the difference in gene expression level between the two treatment groups of broiler chickens and the statistical significance of the difference (Figure 2A).

Hierarchical clustering analysis was applied to compare the gene expression patterns of DEGs in the two treatment groups of broiler chickens (Figure 2B). Different colors in the heatmap represent different expression levels. The highest expression level is represented by the red color while blue represents the lowest expression level. The heatmap results show that the expression patterns of the same group of DEGs were in the same cluster. For example, the low lysine samples (LR1-7233, LR1-7233 and LR1-7224) were highly enriched in several genes (TF, GAPDH, SPP2, APOC3, ENO1, NME4, APOA1, HPD, VTN) that were significantly downregulated whereas the high lysine samples (LR3-7278 and LR3-7345), these same genes were upregulated. Sample clustering results also showed that samples from the same lysine treatment were classified into the same cluster and samples from different lysine treatments were differentiated, which confirmed the reliability of the sampling in this study.

### 2.4. GO Enrichment and KEGG Pathway Analysis of DEGs

DEGs were used for gene ontology (GO) analysis to reveal their functional enrichment in the comparison of the two treatment groups. The DEGs were categorized into three main GO categories: biological process, cellular component and molecular function. In total, there were 210 significantly enriched GO terms (*p* < 0.05) that were identified by comparing the low-lysine and high-lysine treatments. Top significantly enriched biological process terms in this comparison were associated with cell growth and development, biological adhesion, cell communication, drug metabolic process, cell proliferation, regulation of insulin-like growth factor receptor signaling pathway, cell death, response to stress, lipid transport, lipid metabolic process, cellular response to starvation, cellular catabolic process, cellular response to fatty acid, cell development, lipid homeostasis, ion homeostasis, lysine catabolic process and lysine biosynthetic process via aminoadipic acid (Table 3). A full list of summarized significantly enriched lysine related biological processes with GO terms involved in lysine metabolism, lysine transport and lysine modifications is shown in Table S1. These significantly enriched lysine related biological processes are involved in lysine metabolism, lysine transport and lysine modifications. Genes associated with these GO terms might be crucial for muscle development and chicken growth.

**Figure 3:**
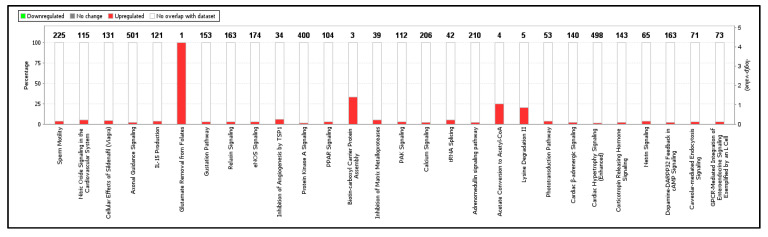
Gene ontology enrichment analysis of differentially expressed transcription factors in the lysine-deficient vs. lysine-adequate treatment groups of broiler chickens. *X*-axis indicates various significantly enriched pathways and number of differentially expressed transcriptional factors; *Y*-axis indicates the percentage of differentially expressed genes. The bars indicate upregulation and downregulation.

Kyoto Encyclopedia of Genes and Genomes (KEGG) enrichment analysis of DEGs showed that 210 genes were annotated in the KEGG database. There were 28 significantly enriched KEGG pathways (*p* < 0.1) in the comparison of low and high lysine treatments (Figure 3). These pathways are mostly related to the metabolism of amino acids, carbohydrates and lipids and disease progression; lysine degradation II; protein kinase signaling; glutamate removal from folates; PPAR signaling; tRNA splicing; acetate conversion to acetyl-CoA and PAK signaling, among others (Figure 3). Some of the top DEGs in these enriched pathways include AASS, TGFB1, ROCK2, PPARGC1A, IGFBP2 and KLF4, among others (Table 4).

### 2.5. Validation of DEGs by qRT-PCR

**Figure 4:**
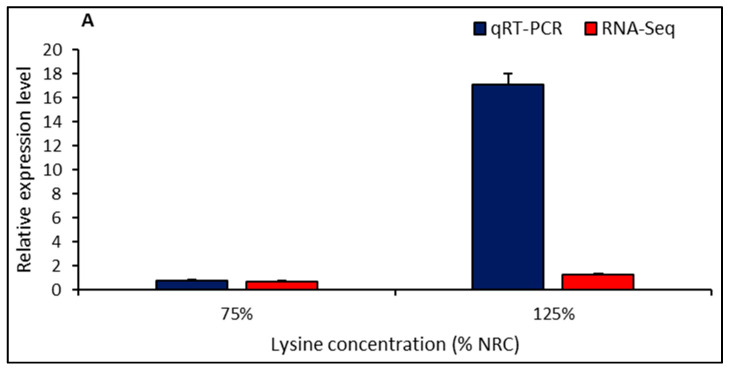

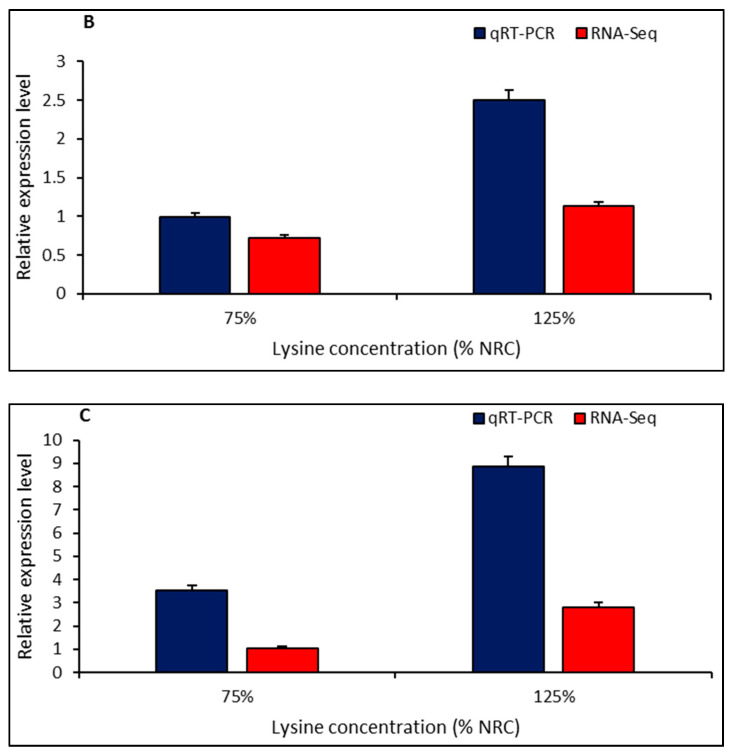
Validation of RNA-seq data. Expression levels of three DEGs ((**A**): SLC7A1; (**B**): SLC7A2; (**C**): SLC7A3) in the liver tissues of high- and low-lysine-fed broiler chickens. qRT-PCR: quantitative real-time polymerase chain reaction; RNA-seq: RNA sequencing. Results from qRT-PCR of the three genes were calculated using the 2^−∆∆*C*t^ method while the RNA-seq expression levels were estimated according to fragments per kilobase of transcripts per million mapped fragments (FPKM).

To validate the RNA-seq expression profiles, three DEGs (SLC7A1, SLC7A2 and SLC7A3) obtained from RNA-seq within the lysine transport pathway were selected for qRT-PCR, using the same RNA samples that were utilized for RNA-seq. The comparative results of the log2-fold changes predicted by RNA-seq and qRT-PCR are shown in Figure 4. The qRT-PCR analysis confirmed that all the selected DEGs were differentially expressed between the chickens fed low-lysine and high-lysine concentration diets. This therefore attests to the reliability and accuracy of our sequencing data.

## 3. Discussion

Lysine is an essential amino acid required for animal growth and maintenance. It is used as a reference amino acid to formulate animal diets because it represents one of the most limiting amino acids in practical corn-soybean meal, sorghum-soybean meal and many other diets for broilers in poultry production. The effects of lysine intake and maximum tolerable lysine intake in broilers have been well documented [27,28,29,30,31,32,33,34,35]. These studies define the maximum tolerable levels of amino acids such as lysine, as recommended by the National Research Council (NRC). This is because the NRC recommendation provides a benchmark that is useful for animal studies on amino acids. Updates of nutrient requirements of poultry are available from the NRC up to 1994, since they were first reported in 1944 [36].

In the broiler industry, varying dietary lysine has been found to greatly alter the gene expression of growing chicks, hence affecting their growth performance [37]. Alterations in mRNA expression levels are reflective of functional changes in the cells, as reported by several researchers [38,39]. In this study, we investigated the role of dietary lysine in the identification of lysine-dependent genes and their molecular mechanisms using transcriptome data. The resultant outcomes can be used as main targets for formulating strategies to improve the growth performance and production of broiler chicken. Our growth performance findings revealed that decreasing dietary lysine significantly decreased growth by 43% and feed intake by 38%, while the FCR increased by 13%. This shows that growth is highly impaired when the concentrations of lysine are significantly reduced, hence causing the deaths of broiler chickens. Similar studies by Nasr and Kheiri [3], Bhogoju et al. [5], Khwatenge et al. [6], Kerr et al. [40] and Corzo et al. [41] have confirmed this effect of lysine on the growth performance of birds. The reduction in feed intake may likely be due to the birds experiencing satiety effects, hence reducing growth [4,11]. This may be explained by the mediation of lysine via neuroendocrine food regulation. Recent studies have shown that dietary lysine concentration may influence signaling pathways that regulate food intake via the brain–liver axis by the neuroendocrine system [6,11].

This study revealed adverse outcomes, such as decreases in the initial body weight, BWG, feed intake and feed efficiency, in chickens fed low lysine concentrations. This finding was consistent with that of Bhogoju et al. [5], who reported that a low (80% NRC) concentration of lysine in diets decreased the BW and feed intake of broilers compared with the high (120% NRC) or NRC-recommended (100%) lysine concentration intake. Nasr and Kheiri [3] also reported that decreasing lysine concentration in feed from 1.14% to 0.8% decreased the body weight, BWG and feed intake of broilers. Therefore, a low lysine intake can adversely affect the growth performance of broiler chickens. Moreover, our results showed that the effects of dietary lysine on broiler performance are concentration-dependent and this cannot be well explained by nutritive experiments alone, because the molecular mechanism associated with lysine uptake are still unclear. For this reason, we analyzed gene expression using RNA-seq in the broiler liver and then aimed to explore the molecular mechanisms of DEGs identified using genomic analysis tools, based on the available literature for poultry and other monogastric animals.

A good number of genes related to growth have been discovered but their specific molecular mechanisms for growth and development are still unclear [42]. In the current study, RNA-seq was carried out in broiler chickens fed low- and high-lysine diets to reveal lysine-dependent genes and other differentially expressed genes that influence growth. A total of 210 differentially expressed genes (DEGs) were identified by comparing the gene expression between the broiler chickens fed low- and high-lysine diets.

Results of our GO enrichment analysis indicate that the downregulated and upregulated genes in the comparison of broilers fed the low- vs. high-lysine diets resulted in identification of the DEGs, which were in turn associated with GO biological process terms mainly involved in cell growth, biological adhesion, developmental processes, cell communication, drug metabolic processes, cell proliferation, regulation of insulin-like growth factor receptor signaling pathway, cell death, response to stress, lipid transport, lipid transport, lipid metabolic process, cellular response to starvation, cellular catabolic process, cellular response to fatty acid, cell development, lipid homeostasis, ion homeostasis, lysine anabolic and catabolic processes, lysine transport, metabolic processes, lysine biosynthetic process via aminoadipic acid, biological adhesion and response to stress. Most of these terms were related to the uptake of fatty acids, carbohydrates and proteins and metabolism. Top detected DEGs in these processes include AASDHPPT, AASS, SLC26A6, FGF1, SLC7A1, SLC7A2, SLC7A3, TGFB1 and PQLC2 (SLC66A1).

Several pathways are related to muscle growth, namely PAK signaling, the peroxisome proliferator-activated receptor (PPAR) signaling pathway and the acetate conversion of Acetyl-CoA signaling pathway, protein kinase A (PKA) signaling. Previous studies have reported the involvement of these pathways in maintaining the normal metabolism of cells, the proliferation and differentiation of muscle cells and promoting muscle fiber formation [42]. Two pathways are related to lysine breakdown and transport, namely lysine degradation II and glutamate removal from folate pathways.

The PPAR pathway was significantly enriched. This pathway is a critical pathway in lipid metabolism. In our studies, the most upregulated gene in the PPAR pathway is the PPARG coactivator 1 alpha (PPARGC1A) gene. PPARGC1, which is highly expressed in the liver tissues, is a transcriptional coactivator that regulates energy metabolism [43,44,45]. The activation of PPARGC1A in the PPAR pathway indicates that this gene is highly involved in cellular metabolism regulation during lysine-adequate and lysine-deficient conditions, as well as adaptation of the birds during stressful conditions such as lack of carbohydrates. Kumar et al. also reported that various genes, including the PPARGC1A gene enriched in the PPAR pathway, play a vital role in energy metabolic pathways to regulate the requirements of chickens in the liver [45].

The KEGG pathway enrichment analysis of the DEGs revealed very important pathways that are involved in amino acid transport as well as the utilization of these amino acids in the cells. Some of these pathways include the lysine signaling pathway, the mTOR signaling pathway and lysine metabolism. Some of the key genes in these pathways that are closely associated with cationic amino acid transport and utilization include SLC7A1, SLC7A2 and SLC7A3. Among these three genes, SLC7A1 was the most highly upregulated gene, showing its abundance and its involvement in the transport of lysine. Analogously, previous studies have also demonstrated that an increase or decrease in the lysine concentration can alter the expression of cationic amino acids and hence affect the uptake of amino acids involved in protein synthesis [46]. Thus, we focused on the effect of lysine on the gene expression of SLC7A1, SLC7A2 and SLC7A3 genes in chicken liver and how lysine intake is regulated by these genes. The expression of SLC7A1, SLC7A2 and SLC7A3 in the liver under different concentrations of lysine shows that these genes were significantly upregulated in a dose-dependent manner. It is known that these amino acid transporters are highly involved in the transport of lysine through the mTORC1-signaling pathway [47,48,49,50,51]. Cationic amino acid transporters, among other amino acids, have been proposed to be extracellular amino acid sensing and transmitting molecules [52,53]. We confirmed this phenomenon through our expression analysis of these genes. Therefore, the ingestion of lysine altered the expression of SLC7A1, SLC7A2 and SLC7A3, as well as other genes associated with amino acid sensing, transport and mTORC1 regulation in the liver, which stimulates protein synthesis via the mTORC1. Therefore, the alteration of dietary lysine contributes to the gene expression of cationic amino acid transport, which may alter the growth patterns of broiler birds through amino acid signaling.

## 4. Materials and Methods

### 4.1. Ethics Statement

All sample collections and treatments were conducted strictly in accordance with the protocol approved by the Institutional Animal Care and Use Committee (IACUC) of Tennessee State University, USA (Assurance # A4472-01, 2016).

### 4.2. Management of Experimental Birds and Dietary Treatments Design

A total of 336 one-day-old broiler chicks were obtained from Aviagen (Huntsville, AL, USA) and assigned to 24 floor pens, where they were raised at Frank A. Young poultry research facility (Tennessee State University, Nashville, TN, USA) up to 8 weeks of age (WOA). Birds were randomly assigned to 3 dietary lysine treatments comprising distinct concentrations for the starter and grower periods. The lysine concentrations were 0.855%, 1.14% and 1.42% for the starter period and 0.75%, 1.00% and 1.25% for the grower period. These diets were 75%, 100% and 125% of the NRC recommended lysine requirement for broiler chickens. The control lysine diets were 1.14% and 1.00% for the starter and grower periods, respectively. The dietary treatments were replicated 8 times, with each replicate housing a total of 14 birds. The starter diets were isocaloric (3100 Kcal ME/kg) and isonitrogenous (23% crude protein) and were fed from 0–4 WOA. The grower diets were also isocaloric (3200 Kcal ME/kg) and isonitrogenous (22% CP) and were fed from 5–8 WOA. Composition of experimental diets is shown in Table S2. All experimental diets containing the various concentrations of supplemental lysine were mixed at the Frank A. Young poultry research facility (Tennessee State University, Nashville, TN). Free amino acid analyses were carried out at the Proteomics & Mass Spectrometry Facility at the Danforth Plant Science Center (St. Louis, MO, USA). The housing temperature was maintained at 32.2 °C for the first week and was gradually reduced by 2.8 °C weekly, to a steady temperature of 23.8 °C. At 4–8 WOA, no supplemental heating was provided to the birds and a constant room temperature was maintained at 21 °C. Ventilation within the growing pens was kept by thermostatically controlled exhaust fans for all birds. Feed was provided in mash form and both feed and water were provided for ad libitum consumption. Body weight and feed consumption (FC) were measured weekly and body weight gain (BWG) and feed conversion ratio (FCR) were calculated weekly using the body weight and feed consumption data. Feed and water troughs were cleaned weekly. Mortality was monitored and recorded as it occurred.

### 4.3. Tissue Sample Collection

At 8 WOA, a total of 69 birds (20% of total), 23 birds from each of the three dietary treatments, was randomly selected and sacrificed via cervical dislocation. Tissue samples were excised from liver and intestines and collected and immediately snap-frozen in liquid nitrogen and stored at −80 °C until use. All the intestine samples were taken from the same part (duodenal loop at about 1 cm from the Meckel’s diverticulum).

### 4.4. Total RNA Extraction and RNA-Seq Library Construction

Total RNA was extracted from the liver using TRIzol™ Reagent (Invitrogen, Life Technologies, Carlsbad, CA, USA), according to the manufacturer’s protocol. The whole transcriptome library was constructed using the Ion Total RNA-Seq Kit v2 (Thermo Fisher Scientific, Waltham, MA, USA) after polyadenylation of total RNA prepared using the Poly(A)Purist™ Kit (Thermo Fisher Scientific, Waltham, MA, USA). The resultant purified cDNA was dissolved in RNase-free water; then, the yield and distribution were assessed. The concentrations of the purified RNA and cDNA were measured using a NanoDrop™ Spectrophotometer (NanoDrop Technologies, Wilmington, DE) and the Qubit RNA Assay and dsDNA HS Assay Kit with the Qubit™ 2.0 Fluorometer, respectively (Life Technologies, CA, USA), by taking the optical density (OD) at 260 nm and 280 nm. The purified RNA and cDNA with A260/A280 ratio above 1.7 were retained [54]. The size distribution of the library was analyzed on the Agilent™ 2100 Bioanalyzer™ instrument, using the Agilent™ DNA 1000 Kit (Agilent Technologies, CA, USA). Samples with RNA integrity numbers (RIN) of 8 and above were retained. Sequencing libraries were amplified on the Ion OneTouch™ 2 instrument, using the Ion PGM™ Hi-Q™ View OT2 Kit (Thermo Fisher Scientific, Waltham, MA, USA), and the template-positive ISPs were recovered and enriched on Ion OneTouch™ ES instrument (Thermo Fisher Scientific, Waltham, MA, USA), according to the manufacturer’s recommendations. The enrichment efficiency was determined using the Guava™ easyCyte™ 5 Flow Cytometer (MilliporeSigma, Billerica, MA, USA). A planned run was prepared, and the Ion 318™ chip was loaded with the enriched, template-positive ISPs, and the sequencing was performed on the Ion PGM™ System installed with Torrent Suite™ Software v5, according to the manufacturer’s protocol.

### 4.5. Quality Control and Comparative Analysis

The sequencing reads obtained were analyzed on CLC Genomics Workbench software ver.12 (CLC bio, Aarhus, Denmark). The quality of the raw data in fastq format was assessed. Low-quality reads containing sequencing adaptors and poly-N, unknown bases and low-quality bases were removed. At the same time, the Q20, Q30, final library percentage, GC content and sequence duplication level of the clean data were calculated. All the downstream analyses were based on high-quality clean data. The clean reads obtained after data filtering were mapped to the chicken reference genome sequence (Galgal5). Only reads with a perfect match or one mismatch were analyzed and annotated based on the reference genome.

### 4.6. Differential Expression Analysis

The quantification of gene expression levels was performed as follows. The gene expression levels were estimated according to fragments per kilobase of transcript per million fragments mapped (FPKM) [55]. Differential expression analysis between the two groups was performed using the CLC genomics workbench. The differential expression data were statistically determined using a model based on the negative binomial distribution. The genes with an adjusted *p*-value ≤ 0.05 and a fold change > 1 were considered to be differentially expressed. Fold change represents the ratio of the expression between the two groups. The resulting *p*-values were adjusted using Benjamini and Hochberg’s approach for controlling the false discovery rate (FDR) [56].

### 4.7. GO and KEGG Pathway Enrichment Analysis

The Gene Ontology database (GO: http://www.geneontology.org/) is a structured, standard biological annotation system built by Gene Ontology Consortium. The aim of the Gene Ontology database is to build three independent ontologies: biological process, molecular function and cellular component [57]. KEGG (https://www.genome.jp/kegg/) is a database resource for understanding the high-level functions and utilities of the biological system, including the cell, the organism and the ecosystem, from molecular level information, especially large scale molecular datasets generated by genome sequencing and other high-throughput experimental technologies [58]. All the target genes of the differentially expressed mRNA were subjected to GO and KEGG pathway enrichment analysis. REViGO (http://revigo.irb.hr/) was used to summarize long lists of GO terms by removing redundant GO terms [59].

### 4.8. Functional Analysis of DEGs Using Ingenuity Pathway Analysis (IPA)

Ingenuity^®^ Pathway Analysis (IPA, Qiagen Inc., Hilden, Germany) was used for the functional annotation and mapping of DEGs to canonical pathways, biological processes and gene interaction networks. IPA Fisher’s exact test is used by IPA to test for significance (*p* ≤ 0.05) and over-representation of DEGs in canonical pathways and biological processes. The DEGs accepted by IPA are considered as analysis-ready if the gene is curated and annotated in the Ingenuity^®^ Knowledge Base, which is mainly curated from the mammalian biomedical literature and devoid of many avian-specific genes. A combination of the chicken gene symbol (primary gene ID) and the NCBI RefSeq Protein ID (secondary gene ID) was used to maximize the number of chicken gene DEGs accepted by IPA as analysis-ready DEGs. The Ingenuity^®^ Upstream Regulator Analysis within IPA was used to identify major transcription factors and to predict either activation or inhibition of direct target genes by their upstream regulators.

### 4.9. Validation of RNA-Seq Results

The expression of 3 genes was quantified by qRT-PCR to validate the RNA-seq data. Primer pairs used for quantification were designed using the National Center for Biotechnology Information’s (NCBI) Primer3^®^ Software (https://www.ncbi.nlm.nih.gov/tools/primer-blast/; Massachusetts Institute of Technology’s Whitehead Institute for Biomedical Research, Cambridge, MA, USA), as shown in Table S3. The primers were designed such that they covered different exons in order to assure the amplification of the cDNA [15]. In the study, β-actin was used as the housekeeping gene [60]. The primers were synthesized by Eurofins Genomics (Louisville, KY, USA). The validation was performed on the treatment samples, with low-, control and high-lysine diets used for the RNA-seq analysis as biological replicates. Total RNA was reverse-transcribed into cDNA using the High-Capacity cDNA Reverse Transcription Kit (Thermo Fisher Scientific, Waltham, MA, USA). qRT-PCR was conducted on an Applied Biosystems PRISM 7000^®^ real-time PCR sequence detection system (Applied Biosystems, Foster City, CA, USA) in a total volume of 20 μL, including 10 μL of QiaGen’s QuantiTect^®^ SYBR^®^ Green PCR Kit (QiaGen Incorporated, Valencia, CA, USA), 1 μL of each primer (10 μM), 6 μL of RNase-free water and 2 μL of cDNA, according to the manufacturer’s protocol.

The PCR efficiency of each primer pair was calculated by the standard curve method, using five points of cDNA serial dilutions. The cDNA template used for this purpose came from a pool of 6 cDNAs from the 6 samples used for RNA-Seq. qRT-PCR assays were programmed at the following cycling parameters: initial denaturation at 95 °C for 15 min, followed by 40 cycles of denaturation at 94 °C for 15 s and primer annealing at 55 or 57 °C for 30 s, and final extension at 72 °C for 30 s. For quality control, a dissociation curve was added. Data were presented as threshold cycle (Ct) relative to an internal control β-actin. To compare the results of qRT-PCR with the sequencing-based results, we transformed the mean 2^−ΔΔCT^ value for each gene into a fold change.

### 4.10. Statistical Analysis

Growth performance data were subjected to analysis of variance (ANOVA) using the general linear model (GLM) of the SAS^®^ software as a completely randomized design with dietary treatments as main effects [61]. All variables were analyzed as repeated measurements. Percentage and gene expression data were transformed into log form prior to analysis. The data were then back-transformed for tabulation and discussion. The statistical model used was Y_ijk_ = µ + T_i_ + R_ij_ + γ_ijk,_ where Y_ijk_ = response variables from each individual replication, µ = the overall mean, T_i_ = the effect of dietary treatment (lysine) s, R_ij_ = the inter-experimental unit (replications) error term, and γ_ijk_ = the intra-experimental unit error term. Differences in mortality among dietary treatments were analyzed using the chi-square method. Least significant difference comparisons were made between treatment means for main effects which had a significant *p*-value. Data were presented as means ± the standard error. Significant results implied that *p* ≤ 0.05. Fold change was calculated using the comparative C_T_ method discussed by Schmittgen and Livak [62,63]. Fold change equals 2^−∆∆C_T_^, where −∆∆C_T_ = (C_T_ gene of interest − C_T_ internal control Sample A) – (C_T_ gene of interest − C_T_ internal control sample B).

## 5. Conclusions

In this study, we demonstrated that an increase in dietary lysine leads to an increase in BWG and feed intake. By using RNA-seq analysis tools, we were able to identify significantly enriched pathways, protein-association networks and co-occurrence analysis of DEGs in the livers of broiler chickens fed different concentrations of lysine. We identified DEGs that are directly related to growth, lysine intake and transport as well as lysine signaling. Our findings therefore contribute to a better understanding of the potential molecular mechanisms underlying the correlation between lysine intake, BWG and feed intake in broiler chickens. Moreover, DEGs obtained in this study may be used as potential candidate genes for further investigation into methods of improving broiler chicken growth. This study therefore provides insights into the molecular mechanisms of bird responses to availability of lysine and may be key in uncovering a significant number of lysine dependent genes.

## Figures and Tables

**Table 1:** Effects of dietary lysine concentration on the growth performance of broiler chickens during the 8-week period.

Parameters	Dietary Lysine Concentration			SEM	*p*-Value
	75%	100%	125%		
Initial body weight, g	39	39	39	0.057	*p* < 0.05
8-week body weight, g	2002 ^c^	3513 ^b^	4180 ^a^	119.2	*p* < 0.05
8-week body weight gain, g	1983 ^c^	3493 ^b^	4147 ^a^	128.7	*p* < 0.05
Feed intake, g/8 weeks	3860 ^c^	6277 ^b^	7132 ^a^	72.6	*p* < 0.05
^1^ Feed conversion ratio	1.98 ^a^	1.75 ^b^	1.63 ^b^	0.09	*p* < 0.05
Lysine consumption, g/8 weeks	32.81 ^c^	71.56 ^b^	101.3 ^a^	19.83	*p* < 0.05
Percentage mortality	21.43 ^a^	3.57 ^b^	2.91 ^b^	-	*p* < 0.05

^a,b,c^ Means with different superscript letters differ significantly at *p* < 0.05. ^1^ Feed conversion ratio was calculated by dividing the feed intake (g) by the body weight gain (g).

**Table 2:** Data summary from RNA-seq. RNA-seq reads and mapping rates of six chicken liver samples fed diets containing different lysine concentrations.

Sample ID	Raw Reads	Clean Reads	Total Bases	Clean Bases	Mean Read Length (bp)	GC Content (%)	Final Library (%)
LR1-7233B	3,668,185	3,165,966	9,941,1210	9,940,3150	144	52.46	86
LR1-7224	3,250,491	2,816,687	444,050,107	395,722,688	158	44.76	87
LR1-7233	1,399,447	644,481	65,085,876	54,302,460	94	52.29	87
LR3-7278	3,795,682	3,186,700	399,444,431	363,388,261	125	48.91	89
LR3-7345	3,627,938	2,804,016	343,715,544	318,661,316	122	48.21	77
LR3-7320	4,876,392	4,241,423	589,052,379	551,541,058	139	49.82	51

**Note:** Clean reads: total number of pair-end reads in the clean data; clean bases: total number of bases in the clean data; GC content: percentage of G and C bases in the clean data; final library: the percentage of library ion sphere particles (ISPs) that pass the polyclonal, low quality, and primer dimer filters; RNA-seq: RNA Sequencing; LR1: Low lysine samples; LR3: High lysine samples.

**Table 3:** Significantly enriched biological processes involved in cell growth, development and lysine transport.

Term ID	Description	Genes	Frequency (%)	Uniqueness
GO:0016049	Cell growth	ALCAM, EXFABP, ID2, LLPH, SEMA5A, SEMA5B, SHH, TTR, ULK1	1.71	0.85
GO:0022610	Biological adhesion	M2, TENM3, TGFBI, THY1, TINAG, TINAGL1, TNFSF8, UBASH3B, VCL, VWA2	5.68	0.99
GO:0032502	Developmental process	SAP, PTK2, PTK7, PTN, PUM1, PXN, PYGO1, RAB10, RAB18, RAB3A, RAB8A, RAC3	21.32	1
GO:0040007	Growth	FGF1, GAP43, ID2, LARGE1, LLPH, PCK1, PSAP, PTK7, SEMA5A, SEMA5B, SHH, TEC, TTR, ULK1	3.21	0.99
GO:0007154	Cell communication	RNF152, RPTOR, RRAGC, SESN1, SESN2, SESN3, SIK2, SLC12A4, SLC12A7, STK11, WDR45B, WIPI2	27.90	0.98
GO:0017144	Drug metabolic process	CYP1A1, CYP1A2	0.05	0.97
GO:0008283	Drug Cell proliferation	ACE, ACVR2A, ACVR2B, BMP4, BMPR1A, CD180, CD40LG, ENTPD5, EXFABP, ID2, TGFB2, TOP2A, TWIST1, USP28, WNT2B, ASCC3	6.48	0.96
GO:0043567	Regulation of insulin-like growth factor receptor signaling pathway	IGFBP1, IGFBP2, IGFBP3, IGFBP4, IGFBP5	0.07	0.88
GO:0008219	Cell death	ATCAY, BNIP2, BRINP1, CASP10, CASP18, CASP2, CASP3, CASP7, CASP8, CASP9, STK4, TGFB2, TGFBR2, TNFRSF21, WNT1, XKR4, XKR9	6.39	0.9
GO:0006950	Response to stress	RPS27A, RPS3, RPTOR, RRAGC, SAMHD1, SELENOK, SEM1, SERP1, SESN1, SESN2, SESN3, SFR1, SGMS1	11.91	0.92
GO:0006869	Lipid transport	SLC43A2, SLC6A9, SLC7A1, SLC7A2, SLC7A3, SLC7A5, SLC7A9	1.42	0.87
GO:0006629	Lipid metabolic process	ACAT1, ACAT2, ACER3, ORMDL2, ORMDL3, OXSM, PCK1, PDCD2	5.34	0.88
GO:0009267	Cellular response to starvation	ATG7, BECN1, BMT2, EIF2S1, GABARAPL1	0.46	0.85
GO:0044248	Cellular catabolic process	ADH5, ADRM1, AFMID, AGL, AGO3, AGO4	5.96	0.93
GO:0071398	Cellular response to fatty acid	EXFABP, PTGER2	0.13	0.87
GO:0048468	Cell development	BMPR1A, CITED2, EXFABP, GDF11, HS6ST1, ID2, INHBA	7.41	0.77
GO:0055088	Lipid homeostasis	ABCG1, ACOX1, ACOX2, EXFABP, LIPG, LPL, MTTP, ORMDL1, ORMDL2, ORMDL3, PLA2G12B, PNPLA2	0.41	0.91
GO:0050801	Ion homeostasis	ANXA5, ANXA6, ANXA7, ATOX1, ATP12A, SLC24A3, SLC30A9, SLC31A1, SLC35G1, SLC9A2, SLC9A7, SLC9A8, SLC9A9	2.62	0.89
GO:0019477	Lysine catabolic process	AASS	1.35	0.91
GO:0019878	Lysine biosynthetic process via Aminoadipic acid	AASDHPPT, AASS	7.50	0.9

**Table 4:** IPA summary of liver transcriptomes in samples from broiler chickens fed diets containing high and low lysine concentrations.

**Top Canonical Pathways**	***p*-Value**	**Overlap**	**Ratio**
Sperm Motility	1.06 × 10^−5^	3.6%	8/225
Nitric Oxide Signaling in the Cardiovascular System	1.65 × 10^−5^	5.2%	6/115
Cellular Effects of Sildenafil (Viagra)	3.73 × 10^−4^	3.8%	5/131
Axonal Guidance signaling	2.39 × 10^−3^	1.6%	8/501
IL-15 Production	2.49 × 10^−3^	3.3%	4/121
**Top Upstream Regulators**	***p*-value of overlap**	**Predicted activation**	
TGFB1	3.63 × 10^−8^	Activated	
ROCK2	5.12 × 10^−7^	-	
PPARGC1A	2.24 × 10^−6^	-	
IGFBP2	2.69 × 10^−6^	Activated	
KLF4	2.73 × 10^−6^	Activated	
**Top Molecular and Cellular Functions**	***p*-value**	**# Genes**	
Cellular Movement	3.87 × 10^−3^–9.67 × 10^−7^	39	
Cellular Assembly and Organization	3.30 × 10^−3^–1.39 × 10^−6^	31	
Cellular Function and Maintenance	3.30 × 10^−3^–1.39 × 10^−6^	37	
Cellular Development	3.49 × 10^−3^–8.20 × 10^−6^	42	
Cellular Growth and Proliferation	3.49 × 10^−3^–8.20 × 10^−6^	33	
**Physiological System Development and Function**	***p*-value range**	**# Genes**	
Organismal Development	3.80 × 10^−3^–2.28 × 10^−7^	58	
Tissue Development and Function	3.80 × 10^−3^–1.12 × 10^−6^	47	
Respiratory System Development and Function	6.94 × 10^−3^–1.85 × 10^−6^	15	
Cardiovascular System Development and Function	3.80 × 10^−3^–6.23 × 10^−6^	43	
Tissue Development	3.80 × 10^−3^–6.50 × 10^−6^	33	
**Top Up-regulated genes**	**Low vs High lysine**	**Top Down-regulated genes**	**Low vs High lysine**
ANO6	8.426	LRRC75B	−7.410
ADAMTS2	8.185	ANKRD22	−7.359
SEMA3C	7.651	ANGPTL4	−3.017
PRKAR2B	7.599	ADAMTS20	−2.87
UPK1B	7.567	MAGI2	−2.43
CYBRD1	7.489	KIF21A	−2.33
ADTRP	7.370	CCDC77	−2.30
ROR2	7.319	ABCC4	−2.20
ITGB6	7.267	CACNA1C	−2.01
PDE1C	7.267	LRP6	−1.56

Ingenuity pathway analysis (IPA) was used for functional analysis of DEGs found in the low lysine vs. high lysine contrast. The top 10 upregulated and downregulated DEGs are presented, along with their respective log2 ratio of treatment conditions.

## Supplementary Materials

Supplementary Materials can be found at https://www.mdpi.com/1422-0067/21/16/5594/s1.

## Abbreviations

DEGs	Differentially expressed genes
SLC	Solute carrier
CATs	Cationic amino acid transports
BWG	Body weight gain
FCR	Feed conversion ratio
FI	Feed intake
FDR	False discovery rate
GO	Gene ontology
KEGG	Kyoto encyclopedia of genes and genomes
qRT-PCR	Quantitative real-time PCR
IPA	Ingenuity pathway analysis
RNA-Seq	RNA sequencing
FPKM	Fragments per kilobase of exon per million fragments
