# Supplementary material for: Transcriptome Analysis and Expression of Selected Cationic Amino Acid Transporters in the Liver of Broiler Chicken Fed Diets with Varying Concentrations of Lysine
Source: Int J Mol Sci. 2020 Aug 5;21(16):5594. doi: 10.3390/ijms21165594 (PMC7460557; doi:10.3390/ijms21165594)
Supplement: Supplementary file 1 [file ijms-21-05594-s001.pdf]

Table S1. Significantly enriched lysine related biological processes with GO terms involved in lysine metabolism, lysine transport and lysine modifications.

GO term	Description	Detected Genes	Detected Genes (Names)
0006553	Lysine metabolic process	2	AASDHPPT, AASS
0006554	Lysine catabolic process	1	AASS
0008612	Peptidyl-lysine modification to Peptidyl-hypusine	1	DOHH
0009085	Lysine biosynthetic process	2	AASDHPPT, AASS
0009086	Methionine biosynthetic process	5	APIP, ENOPH1, MTAP, MTHFR, MTRR
0015819	Lysine transport	1	PQLC2
0017185	Peptidyl-lysine hydroxylation	2	JMJD6, PLOD1
0018022	Peptidyl-lysine methylation	11	ASH2L, DPY30, EEF1AKMT1, SETD3, SETD4, SETD6, SMYD2, SUV39H2, WDR5, WDR61, WDR82
0018023	Peptidyl-lysine trimethylation	4	SETD3, SETD4, WDR61, WDR82
0018026	Peptidyl-lysine monomethylation	4	SETD3, SETD4, SETD6, SMYD2
0018027	Peptidyl-lysine dimethylation	2	SETD3, SMYD2
0018057	Peptidyl-lysine oxidation	2	LOXL2, LOXL4
0018205	Peptidyl-lysine modification	51	ACTL6A, ACTL6B, ASH2L, DOHH, DPY30, EEF1AKMT1, ING3, JMJD6, KANSL2, KANSL3, KAT14, KAT6A, KAT6B, KAT7, LOXL2, LOXL4, MEAF6, MORF4L1, MRGBP, MSL2, MSL3, NAA50, PHF20, PLOD1, RUVBL2, SAE1, SENP1, SENP2,

			SENP5, SENP6, SENP7, SETD3, SETD4, SETD6, SGF29, SIRT5, SMYD2, SUMO1, SUMO2, SUMO3, SUPT7L, SUV39H2, TADA1, TADA3, TAF12, TRIM1BL, UBA2, UBE2I, WDR5, WDR61, WDR82
0018393	Internal peptidyl-lysine acetylation	22	ACTL6A, ACTL6B, ING3, KANSL2, KANSL3, KAT14, KAT6A, KAT6B, KAT7, MEAF6, MORF4L1, MRGBP, MSL2, MSL3, NAA50, PHF20, RUVBL2, SGF29, SUPT7L, TADA1, TADA3, TAF12
0018394	Peptidyl-lysine acetylation	22	ACTL6A, ACTL6B, ING3, KANSL2, KANSL3, KAT14, KAT6A, KAT6B, KAT7, MEAF6, MORF4L1, MRGBP, MSL2, MSL3, NAA50, PHF20, RUVBL2, SGF29, SUPT7L, TADA1, TADA3, TAF12
0018395	Peptidyl-lysine hydroxylation to 5-hydroxy-L-lysine	1	JMJD6
0019477	L-lysine catabolic process	1	AASS
0019878	Lysine biosynthetic process via aminoadipic acid	2	AASDHPPT, AASS
0034968	Histone lysine methylation	7	ASH2L, DPY30, SETD3, SUV39H2, WDR5, WDR61, WDR82
0036047	Peptidyl-lysine demalonylation	1	SIRT5
0036049	Peptidyl-lysine desuccinylation	1	SIRT5
0046440	L-lysine metabolic process	1	AASS
0046485	Ether lipid metabolic process	4	DHRS7B, FAR1, PDCD2, TMEM86A

0070076	Histone lysine demethylation	6	JMJD1C, KDM3A, KDM3B, KDM5B, KDM8, RIOX1
2000756	Regulation of peptidyl-lysine acetylation	4	BRCA1, CEBPB, NOC2L, TADA2A
2000757	Negative regulation of peptidyl-lysine acetylation	2	BRCA1, NOC2L
2000758	Positive regulation of peptidyl-lysine acetylation	3	BRCA1, CEBPB, TADA2A
2001173	Regulation of histone H2B conserved C-terminal lysine ubiquitination	1	WDR70

Table S2. Composition of experimental diets in starter (0-4 WOA) and grower period, 5-8 WOA.

Age (weeks)	0-4			5-8		
Lysine Concentration (%)	0.85	1.14	1.425	0.75	1.00	1.08
Ingredients	%					
Corn, yellow # 2 (8% CP)	50.61	49.22	50.33	61.67	62.02	53.14
Soybean meal (48% CP)	14.10	30.92	31.20	13.50	27.12	34.90
Corn Gluten Meal (60% CP)	17.60	6.00	6.00	17.80	0.00	0.00
Wheat middlings	10.36	4.40	2.67	0.00	0.00	0.00
Alfalfa Meal (17% CP)	1.00	1.00	1.00	1.00	1.00	1.00
Poultry blended fat	2.30	4.62	4.62	2.10	5.50	6.80
Dicalcium Phosphate (18% P, 22% Ca)	1.74	1.74	1.74	1.75	1.75	1.74
Limestone flour (38.8 % Ca)	1.45	1.45	1.45	1.25	1.40	1.45
Salt	0.30	0.30	0.30	0.30	0.30	0.30
Vitamin-Mineral premix[1]	0.25	0.25	0.25	0.25	0.25	0.25
DL-methionine (98%)[2]	0.03	0.01	0.16	0.01	0.16	0.13
L-Arg,%	0.20	0.10	0.28	0.23	0.29	0.20
L-Thr,%	0.00	0.00	0.00	0.12	0.15	0.00
L-Ile,%	0.00	0.00	0.00	0.00	0.00	0.00

L-Val,%	0.00	0.00	0.00	0.02	0.02	0.00
L-Lys HCL,%	0.06	0.00	0.36	0.00	0.04	0.09
Calculated levels						
Metabolizable Energy (Kcal/kg diet)	3,100	3,100	3,100	3,200	3,200	3,200
Crude Protein	23	23	23	22	22	22
L-Lys	0.85	1.14	1.42	0.75	1.00	1.08
Analyzed levels						
ME, Kcal/kg	3,100	3,100	3,100	3,200	3,200	3,200
CP, %	23	23	23	22	22	22

[1] Provided per kg of diet: retinyl acetate,3, 500 IU; cholecalciferol,1,000 ICU; DL-α-tocopheryl acetate,4.5 IU; menadione sodium bisulfite complex, 2.8 mg; vitamin B_{12}, 5.0 mg;riboflavin,2.5 mg; pantothenic acid,4.0 mg;niacin,15.0 mg;choline,172 mg; folic acid, 230 mg; ethoxyquin, 56.7 mg; manganese, 65 mg; iodine,1 mg; iron, 54.8 mg; copper, 6 mg; zinc,55 mg; selenium, 0.3 mg.

[2] Degussa Corporation, Kennesaw, GA.

Table S3. Sequences of primers used in the amplification of CAT-1, CAT-2, CAT-3 and β-actin mRNA of chicken broiler fed diets containing varying concentrations of lysine from hatch to 8 weeks of age.

Gene name and Reference	Primer Sequence [1]	Amplicons length (bp)	Product length (bp)	Tm[2] (⁰C)
CAT-1 (Humphrey et al., 2006)	Forward: 5′-ATGTAGGTTGGGATGGAGCC	20	280	62.4
	Reverse: 3′-AACGAGTAAGCCAGGAGGGT	20	280	62.4
CAT-2 (Humphrey et al., 2006)	Forward: 5′-GTTTCCTTCCTCATTGCTGC	20	200	60.4
	Reverse: 3′-CCACTCCAGGCTCTTGCTAC	20	200	64.5
CAT-3 (Humphrey et al., 2006)	Forward: 5′-CAAGACTGGCTCTGCCTACC	20	236	64.5
	Reverse: 3′-GGATCAACGCAAAGAAGTCC	20	236	60.4
β-actin[3] (Humphrey et al., 2006)	Forward: 5′-CTGACACCACACTTTCTACAATG	24	350	61.0
	Reverse: 3′-GATCTTCATGAGGTAGTCCGTCAG	23	350	64.6

[1] Chicken primers for CAT-1, CAT-2 and CAT-3 genes.
[2] Tm represents the optimized primer annealing temperature.
[3] The β-actin was used as internal controls.
Abbreviations: bp, base pairs; CAT, cationic amino acid transporter.
